# Series: Practical guidance to qualitative research. Part 4: Trustworthiness and publishing

**DOI:** 10.1080/13814788.2017.1375092

**Published:** 2017-12-05

**Authors:** Irene Korstjens, Albine Moser

**Affiliations:** a Faculty of Health Care, Research Centre for Midwifery Science, Zuyd University of Applied Sciences, Maastricht, The Netherlands;; b Faculty of Health Care, Research Centre Autonomy and Participation of Chronically Ill People, Zuyd University of Applied Sciences, Heerlen, The Netherlands;; c Faculty of Health, Medicine and Life Sciences, Department of Family Medicine, Maastricht University, Maastricht, The Netherlands

**Keywords:** General practice/family medicine, general, qualitative designs and methods, trustworthiness, reflexivity, publishing

## Abstract

In the course of our supervisory work over the years we have noticed that qualitative research tends to evoke a lot of questions and worries, so-called frequently asked questions (FAQs). This series of four articles intends to provide novice researchers with practical guidance for conducting high-quality qualitative research in primary care. By ‘novice’ we mean Master’s students and junior researchers, as well as experienced quantitative researchers who are engaging in qualitative research for the first time. This series addresses their questions and provides researchers, readers, reviewers and editors with references to criteria and tools for judging the quality of qualitative research papers. The first article provides an introduction to this series. The second article focused on context, research questions and designs. The third article focused on sampling, data collection and analysis. This fourth article addresses FAQs about trustworthiness and publishing. Quality criteria for all qualitative research are credibility, transferability, dependability, and confirmability. Reflexivity is an integral part of ensuring the transparency and quality of qualitative research. Writing a qualitative research article reflects the iterative nature of the qualitative research process: data analysis continues while writing. A qualitative research article is mostly narrative and tends to be longer than a quantitative paper, and sometimes requires a different structure. Editors essentially use the criteria: is it new, is it true, is it relevant? An effective cover letter enhances confidence in the newness, trueness and relevance, and explains why your study required a qualitative design. It provides information about the way you applied quality criteria or a checklist, and you can attach the checklist to the manuscript.

Key points on trustworthiness and publishing

- The quality criteria for all qualitative research are credibility, transferability, dependability, and confirmability.
- In addition, reflexivity is an integral part of ensuring the transparency and quality of qualitative research.
- Writing a qualitative article reflects the iterative nature of the qualitative research process: continuous data analysis continues with simultaneous fine-tuning.
- Editors essentially use the criteria: is it new, is it true, and is it relevant?
- An effective cover letter enhances confidence in the newness, trueness and relevance, and explains why your study required a qualitative design.

## Introduction

This article is the fourth and last in a series of four articles aiming to provide practical guidance for qualitative research. In an introductory paper, we have described the objective, nature and outline of the series [1]. Part 2 of the series focused on context, research questions and design of qualitative research [2], whereas Part 3 concerned sampling, data collection and analysis [3]. In this paper Part 4, we address frequently asked questions (FAQs) about two overarching themes: trustworthiness and publishing.

## Trustworthiness

### What are the quality criteria for qualitative research?

The same quality criteria apply to all qualitative designs, including the ‘big three’ approaches. Quality criteria used in *quantitative* research, e.g. internal validity, generalizability, reliability, and objectivity, are not suitable to judge the quality of qualitative research. Qualitative researchers speak of trustworthiness, which simply poses the question ‘Can the findings to be trusted?’ [4]. Several definitions and criteria of trustworthiness exist (see Box 1) [2], but the best-known criteria are credibility, transferability, dependability, and confirmability as defined by Lincoln and Guba [4].

Box 1. Trustworthiness: definitions of quality criteria in qualitative research. Based on Lincoln and Guba [4].

**Table:** 

Credibility	The confidence that can be placed in the truth of the research findings. Credibility establishes whether the research findings represent plausible information drawn from the participants’ original data and is a correct interpretation of the participants’ original views.
Transferability	The degree to which the results of qualitative research can be transferred to other contexts or settings with other respondents. The researcher facilitates the transferability judgment by a potential user through thick description.
Dependability	The stability of findings over time. Dependability involves participants’ evaluation of the findings, interpretation and recommendations of the study such that all are supported by the data as received from participants of the study.
Confirmability	The degree to which the findings of the research study could be confirmed by other researchers. Confirmability is concerned with establishing that data and interpretations of the findings are not figments of the inquirer’s imagination, but clearly derived from the data.
Reflexivity	The process of critical self-reflection about oneself as researcher (own biases, preferences, preconceptions), and the research relationship (relationship to the respondent, and how the relationship affects participant’s answers to questions).

### What is credibility and what strategies can be used to ensure it?

Credibility is the equivalent of internal validity in quantitative research and is concerned with the aspect of truth-value [4]. Strategies to ensure credibility are prolonged engagement, persistent observation, triangulation and member check (Box 2). When you design your study, you also determine which of these strategies you will use, because not all strategies might be suitable. For example, a member check of written findings might not be possible for study participants with a low level of literacy. Let us give an example of the possible use of strategies to ensure credibility. A team of primary care researchers studied the process by which people with type 2 diabetes mellitus try to master diabetes self-management [6]. They used the grounded theory approach, and their main finding was an explanatory theory. The researchers ensured credibility by using the following strategies.

Box 2. Definition of strategies to ensure trustworthiness in qualitative research. Based on Lincoln and Guba [4]; Sim and Sharp [5].

**Table:** 

Criterion	Strategy	Definition
Credibility	Prolonged engagement	Lasting presence during observation of long interviews or long-lasting engagement in the field with participants. Investing sufficient time to become familiar with the setting and context, to test for misinformation, to build trust, and to get to know the data to get rich data.
	Persistent observation	Identifying those characteristics and elements that are most relevant to the problem or issue under study, on which you will focus in detail.
	Triangulation	Using different data sources, investigators and methods of data collection. • *Data triangulation* refers to using multiple data sources in time (gathering data in different times of the day or at different times in a year), space (collecting data on the same phenomenon in multiples sites or test for cross-site consistency) and person (gathering data from different types or level of people e.g. individuals, their family members and clinicians). • *Investigator triangulation* is concerned with using two ore researchers to make coding, analysis and interpretation decisions. • *Method triangulation* means using multiple methods of data collection.
	Member check	Feeding back data, analytical categories, interpretations and conclusions to members of those groups from whom the data were originally obtained. It strengthens the data, especially because researcher and respondents look at the data with different eyes.
Transferability	Thick description	Describing not just the behaviour and experiences, but their context as well, so that the behaviour and experiences become meaningful to an outsider.
Dependability and confirmability	Audit trail	Transparently describing the research steps taken from the start of a research project to the development and reporting of the findings. The records of the research path are kept throughout the study.
Reflexivity	Diary	Examining one’s own conceptual lens, explicit and implicit assumptions, preconceptions and values, and how these affect research decisions in all phases of qualitative studies.

*Prolonged engagement*. Several distinct questions were asked regarding topics related to mastery. Participants were encouraged to support their statements with examples, and the interviewer asked follow-up questions. The researchers studied the data from their raw interview material until a theory emerged to provide them with the scope of the phenomenon under study.

*Triangulation*. Triangulation aims to enhance the process of qualitative research by using multiple approaches [7]. Methodological triangulation was used by gathering data by means of different data collection methods such as in-depth interviews, focus group discussions and field notes. Investigator triangulation was applied by involving several researchers as research team members, and involving them in addressing the organizational aspects of the study and the process of analysis. Data were analysed by two different researchers. The first six interviews were analysed by them independently, after which the interpretations were compared. If their interpretations differed, they discussed them until the most suitable interpretation was found, which best represented the meaning of the data. The two researchers held regular meetings during the process of analysis (after analysing every third data set). In addition, regular analytical sessions were held with the research team. Data triangulation was secured by using the various data sets that emerged throughout the analysis process: raw material, codes, concepts and theoretical saturation.

*Persistent observation*. Developing the codes, the concepts and the core category helped to examine the characteristics of the data. The researchers constantly read and reread the data, analysed them, theorized about them and revised the concepts accordingly. They recoded and relabelled codes, concepts and the core category. The researchers studied the data until the final theory provided the intended depth of insight.

*Member check*. All transcripts of the interviews and focus group discussions were sent to the participants for feedback. In addition, halfway through the study period, a meeting was held with those who had participated in either the interviews or the focus group discussions, enabling them to correct the interpretation and challenge what they perceived to be ‘wrong’ interpretations. Finally, the findings were presented to the participants in another meeting to confirm the theory.

### What does transferability mean and who makes a ‘transferability judgement’?

Transferability concerns the aspect of applicability [4]. Your responsibility as a researcher is to provide a ‘thick description’ of the participants and the research process, to enable the reader to assess whether your findings are transferable to their own setting; this is the so-called transferability judgement. This implies that the reader, not you, makes the transferability judgment because you do not know their specific settings.

In the aforementioned study on self-management of diabetes, the researchers provided a rich account of descriptive data, such as the context in which the research was carried out, its setting, sample, sample size, sample strategy, demographic, socio-economic, and clinical characteristics, inclusion and exclusion criteria, interview procedure and topics, changes in interview questions based on the iterative research process, and excerpts from the interview guide.

### What is the difference between dependability and confirmability and why is an audit trail needed?

Dependability includes the aspect of consistency [4]. You need to check whether the analysis process is in line with the accepted standards for a particular design. Confirmability concerns the aspect of neutrality [4]. You need to secure the inter-subjectivity of the data. The interpretation should not be based on your own particular preferences and viewpoints but needs to be grounded in the data. Here, the focus is on the interpretation process embedded in the process of analysis. The strategy needed to ensure dependability and confirmability is known as an audit trail. You are responsible for providing a complete set of notes on decisions made during the research process, research team meetings, reflective thoughts, sampling, research materials adopted, emergence of the findings and information about the data management. This enables the auditor to study the transparency of the research path.

In the aforementioned study of diabetes self-management, a university-based auditor examined the analytical process, the records and the minutes of meetings for accuracy, and assessed whether all analytical techniques of the grounded theory methodology had been used accordingly. This auditor also reviewed the analysis, i.e. the descriptive, axial and selective codes, to see whether they followed from the data (raw data, analysis notes, coding notes, process notes, and report) and grounded in the data. The auditor who performed the dependability and confirmability audit was not part of the research team but an expert in grounded theory. The audit report was shared with all members of the research team.

### Why is reflexivity an important quality criterion?

As a qualitative researcher, you have to acknowledge the importance of being self-aware and reflexive about your own role in the process of collecting, analysing and interpreting the data, and in the pre-conceived assumptions, you bring to your research [8]. Therefore, your interviews, observations, focus group discussions and all analytical data need to be supplemented with your reflexive notes. In the aforementioned study of diabetes self-management, the reflexive notes for an interview described the setting and aspects of the interview that were noted during the interview itself and while transcribing the audio tape and analysing the transcript. Reflexive notes also included the researcher’s subjective responses to the setting and the relationship with the interviewees.

## Publishing

### How do I report my qualitative study?

The process of writing up your qualitative study reflects the iterative process of performing qualitative research. As you start your study, you make choices about the design, and as your study proceeds, you develop your design further. The same applies to writing your manuscript. First, you decide its structure, and during the process of writing, you adapt certain aspects. Moreover, while writing you are still analysing and fine-tuning your findings. The usual structure of articles is a structured abstract with subheadings, followed by the main text, structured in sections labelled Introduction-Methods-Results-Discussion. You might apply this structure loosely, for example renaming Results as Findings, but sometimes your specific study design requires a different structure. For example, an ethnographic study might use a narrative abstract and then start by describing a specific case, or combine the Findings and Discussion sections. A qualitative article is usually much longer (5000–7000 words) than quantitative articles, which often present their results in tables. You might present quantified characteristics of your participants in tables or running text, and you are likely to use boxes to present your interview guide or questioning route, or an overview of the main findings in categories, subcategories and themes. Most of your article is running text, providing a balanced presentation. You provide a thick description of the participants and the context, transparently describe and reflect on your methods, and do justice to the richness of your qualitative findings in reporting, interpreting and discussing them. Thus, the Methods and Findings sections will be much longer than in a quantitative paper.

The difference between reporting quantitative and qualitative research becomes most visible in the Results section. Quantitative articles have a strict division between the Results section, which presents the evidence, and the Discussion section. In contrast, the Findings section in qualitative papers consists mostly of synthesis and interpretation, often with links to empirical data. Quantitative and qualitative researchers alike, however, need to be concise in presenting the main findings to answer the research question, and avoid distractions. Therefore, you need to make choices to provide a comprehensive and balanced representation of your findings. Your main findings may consist, for example, of interpretations, relationships and themes, and your Findings section might include the development of a theory or model, or integration with earlier research or theory. You present evidence to substantiate your analytic findings. You use quotes or citations in the text, or field notes, text excerpts or photographs in boxes to illustrate and visualize the variety and richness of the findings.

Before you start preparing your article, it is wise to examine first the journal of your choice. You need to check its guidelines for authors and recommended sources for reference style, ethics, etc., as well as recently accepted qualitative manuscripts. More and more journals also refer to quality criteria lists for reporting qualitative research, and ask you to upload the checklist with your submission. Two of these checklists are available at http://www.equator-network.org/reporting-guidelines.

### How do I select a potential journal for publishing my research?

Selecting a potential journal for publishing qualitative articles is not much different from the procedure used for quantitative articles. First, you consider your potential public and the healthcare settings, health problems, field, or research methodology you are focusing on. Next, you look for journals in the Journal Citation Index of Web of Science, consult other researchers and study the potential journals’ aims, scopes, and author guidelines. This also enables you to find out how open these journals are to publishing qualitative research and accepting articles with different designs, structures and lengths. If you are unsure whether the journal of your choice would accept qualitative research, you might contact the Editor in Chief. Lastly, you might look in your top three journals for qualitative articles, and try to decide how your manuscript would fit in. The author guidelines and examples of manuscripts will support you during your writing, and your top three offers alternatives in case you need to turn to another journal.

### What are the journal editors’ considerations in accepting a qualitative manuscript?

Your article should effectively present high-quality research and should adhere to the journal’s guidelines. Editors essentially use the same criteria for qualitative articles as for quantitative articles: Is it new, it is true, is it relevant? However, editors may use—implicitly or explicitly—the level-of-evidence pyramid, with qualitative research positioned in the lower ranks. Moreover, many medical journal editors will be more familiar with quantitative designs than with qualitative work.

Therefore, you need to put some extra effort in your cover letter to the editor, to enhance their confidence in the newness, trueness and relevance, and the quality of your work. It is of the utmost importance that you explain in your cover letter why your study required a qualitative design, and probably more words than usual. If you need to deviate from the usual structure, you have to explain why. To enhance confidence in the quality of your work, you should explain how you applied quality criteria or refer to the checklist you used (Boxes 2 and 3). You might even attach the checklist as additional information to the manuscript. You might also request that the Editor-in-Chief invites at least one reviewer who is familiar with qualitative research.

Box 3. Quality criteria checklists for reporting qualitative research. Based on O’Brien et al. [9]; Tong et al. [10].

**Table:** 

Standards for reporting qualitative research (SRQR)	Consolidated criteria for reporting qualitative research (COREQ)
All aspects of qualitative studies.	Qualitative studies focusing on in-depth interviews and focus groups.
21 items for: title, abstract, introduction, methods, results/findings, discussion, conflicts of interest, and funding.	32 items for: research team and reflexivity, study design, data analysis, and reporting.
